# MiRNA-30e downregulation increases cancer cell proliferation, invasion and tumor growth through targeting RPS6KB1

**DOI:** 10.18632/aging.203665

**Published:** 2021-11-02

**Authors:** Lin Wang, Xiang-Bo Ji, Li-Hong Wang, Zhong-Kun Xia, Yun-Xia Xie, Wen-Jing Liu, Jian-Ge Qiu, Bing-Hua Jiang, Ling-Zhi Liu

**Affiliations:** 1 BGI College and Henan Institute of Medical and Pharmaceutical Sciences, Zhengzhou University, Zhengzhou, China; 2 Academy of Medical Science, Zhengzhou University, Zhengzhou, China; 3 Department of Pathology, Anatomy and Cell Biology, Thomas Jefferson University, Philadelphia, PA 19107, USA; 4 Department of Medical Oncology, Thomas Jefferson University, Philadelphia, PA 19107, USA

**Keywords:** miR-30e, RPS6KB1, tumor growth, esophageal carcinoma, clinical outcome

## Abstract

Human esophagus carcinoma (EC) is one of the most common malignant tumors, especially in Africa and Asia including China. In EC initiation and progression, genetic and epigenetic aberrations have been reported to play a major role, but the underlying molecular mechanisms are largely unknown. In this study, the miR-30e levels were analyzed in human EC tissues and TCGA databases, and the results demonstrated that miR-30e expression in EC tissues was significantly decreased compared to adjacent normal tissues. To further investigate the role of miR-30e in cancer cells, we found that forced expression of miR-30e dramatically inhibited cell proliferation, invasion, tube formation, and colony formation of cancer cells. To determine the underlying mechanism of miR-30e, we found that RPS6KB1 was a direct target of miR-30e by binding to its 3′-UTR, which was verified by luciferase activity assay using reporters with wild-type miR-30e and its seed sequence mutant constructs and Western blotting assay. *In vivo* experiment showed that miR-30e overexpression significantly inhibited tumor growth and decreased RPS6KB1 expression in xenografts. In EC, high expression of RPS6KB1 in tumor tissues indicated poor prognosis of patients with less survival rate. High levels of RPS6KB1 and low levels of miR-30e closely correlated poor survival of patients with several other types of cancer. These findings show that miR-30e and its target RPS6KB1 are important in cancer development and clinical outcomes, and miR-30e/RPS6KB1 is a potential future therapeutic pathway for EC intervention.

## INTRODUCTION

Esophageal cancer (EC) is one of the most frequently reported cancer-related deaths worldwide and its incidence in Asian countries, such as certain areas of China, has increased sharply during recent decades [1, 2]. Adenocarcinoma and squamous cell carcinoma are two distinct subtypes of EC [3]. Despite recent progress in EC diagnostic and therapeutic strategies such as surgery combined with chemotherapy or radiation therapy, the overall 5-year survival rate for EC patients remains quite low (10–20%) due to high systemic toxicity and drug resistance to cancer treatment [4–6]. Recent studies have reported that the major drivers of EC carcinogenesis and progression are abnormal functions of the oncogenes or tumor suppressors [7, 8]. However, details of the molecular mechanism for EC development are still unclear. Thus, further study in molecular basis of EC is imperative to support the development of new therapeutic strategies to improve clinical survival rate of EC patients.

MicroRNAs are a group of small non-coding RNA molecules, typically 18 to 24 nucleotides, that regulate gene expression by targeting 3′-untranslated regions (UTRs) of potential gene mRNAs [9–11]. Multiple studies have convincingly shown that miRNAs play important roles in biological processes such as inflammation, cell cycle, stress response, differentiation, apoptosis and migration [12–14]. MiRNAs have been observed to be aberrantly expressed in cancers and are involved in the EC gene regulation [9, 15–18]. In addition, miR-30e has been found to be downregulated in several cancers including breast, hepatocellular and bladder cancer [19–21], but the mechanism and role of miR-30e in EC remains to be elucidated.

The mitogen-activated serine/threonine-protein-kinase ribosomal protein S6 kinase B1 (RPS6KB1), also known as the S6K1, is associated with a variety of cellular processes including glucose homeostasis, mRNA processing, protein synthesis, cell proliferation, growth response and other cellular signal transductions [22–24]. RPS6KB1 is activated by serine or threonine residue phosphorylation at threonine 389, and most of its substrates contain a consensus phosphorylation motif (K/RXRXX/T), which is critical to its catalytic activity. In human malignancies, the commonly observed constitutive activation of RPS6KB1 indicates its potential therapeutic role. In addition, Michele Pagano et al. showed that protein kinase S6K1 rapidly phosphorylated the tumor suppressor programmed cell death protein 4 (PDCD4) at Ser67 and subsequently degraded PDCD4 via the ubiquitin ligase SCF (betaTRCP) in response to mitogens [25]. Furthermore, RPS6KB1-mediated Gli1 at Ser84 (downstream hedgehog pathway effector) phosphorylation promoted esophageal cancer development [26]. These results strongly indicate that RPS6KB1 is a potential biomarker for cancer diagnosis and an advantageous target for cancer treatment.

In this study, we plan to address: (a) whether miR-30e expression is associated with EC incidence; (b) what direct target of miR-30e is involved in EC; (c) what roles miR-30e and its target have in cancer cell proliferation and tumor growth; and (d) whether miR-30e/RPS6KB1 axis is associated with overall survival rate in EC cancer patients. The results of this study will be helpful to further understand the molecular mechanisms of EC carcinogenesis and provide potential targets for EC treatment in the future.

## RESULTS

### MiR-30e expression was downregulated in human esophageal carcinoma tissues compared to adjacent normal esophageal tissues

**Figure 1:**
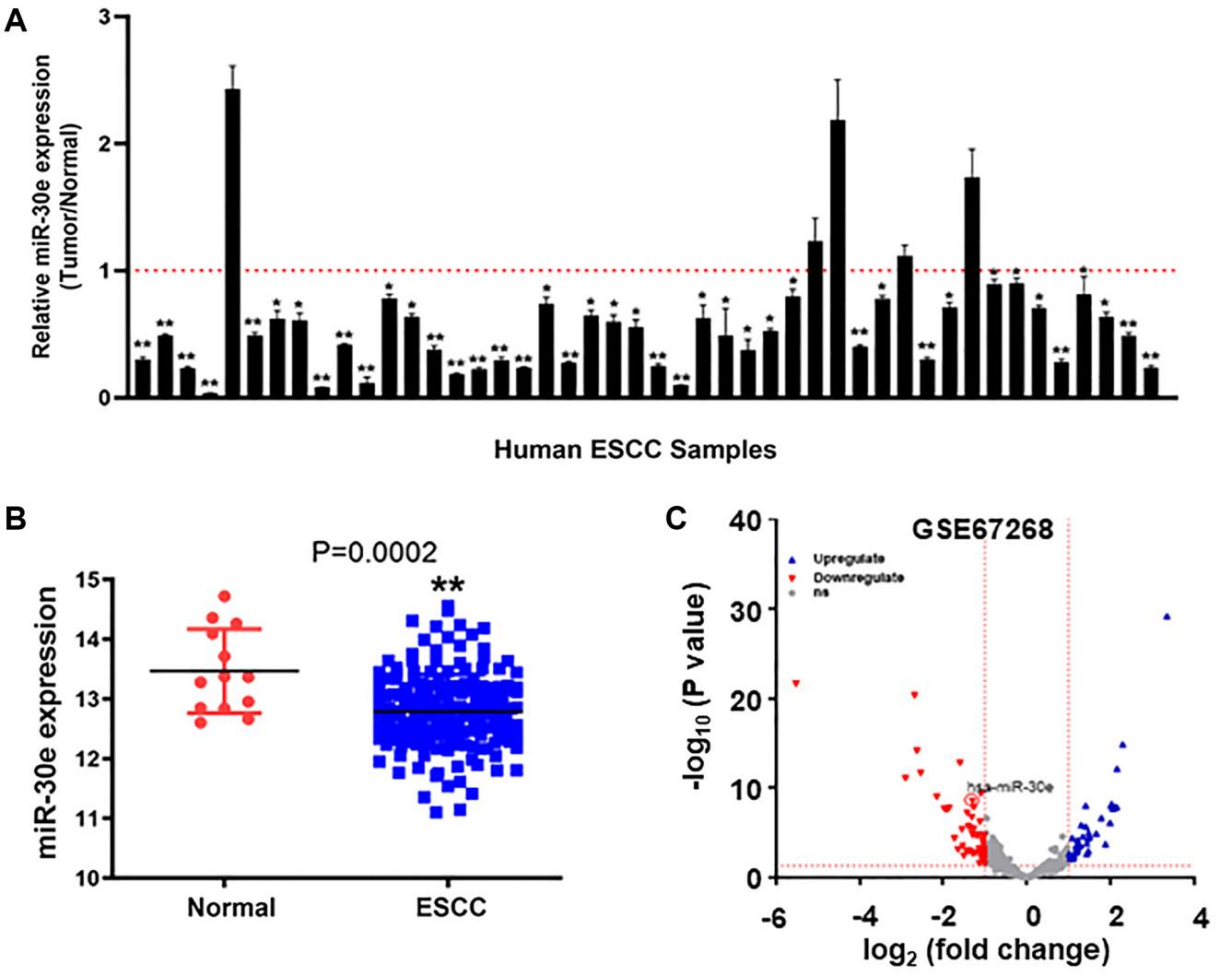
**MiR-30e expression was downregulated in human esophageal carcinoma tissues compared to normal adjacent esophageal tissues.** (**A**) The relative expression levels of miR-30e were detected by qRT-PCR and normalized to an endogenous control (U6 RNA) in 46 pairs of human esophageal carcinoma tissues. The results represent the ratio of miR-30e levels in tumor tissues to adjacent normal tissues. (**B**) Relative expression levels of miR-30e in of 185 human esophageal carcinoma tissues were compared with 13 normal tissues in TCGA database (https://xenabrowser.net/datapages/). (**C**) The miR-30e expression levels of adjacent normal esophageal tissues and esophageal carcinoma tissues were analyzed in the public GEO dataset (GSE67268, https://www.ncbi.nlm.nih.gov/geo/). Data represent mean ± SD of 3 replicates. ^*^indicates significant difference at *P* < 0.05; ^**^indicates significant difference at *P* < 0.01.

MiR-30e was reported to function as a tumor suppressor in some cancers, but its expression level and role in human esophageal carcinoma are not clear yet. We analyzed expression levels of miR-30e in 46 pairs of human esophageal carcinoma tissues and their corresponding non-cancerous adjacent esophageal tissues, and found that miR-30e levels were significantly decreased in human esophageal carcinoma tissues compared to those of adjacent normal tissues (Figure 1A). We also analyzed relative expression levels of miR-30e in 185 human esophageal carcinoma tissues and 13 normal tissues in TCGA database (https://xenabrowser.net/datapages/), and the results also showed significantly downregulation of miR-30e in human esophageal carcinoma tissues compared to normal tissues (Figure 1B). To further confirm the results, we used the GEO dataset to study miR-30e expression levels in esophageal carcinoma tissues and adjacent normal esophageal tissues (GSE67268, https://www.ncbi.nlm.nih.gov/geo/), and found that miR-30e levels were also decreased by 20-fold in human esophageal carcinoma tissues compared to normal adjacent esophageal tissues (Figure 1C).

### MiR-30e overexpression inhibited cell proliferation, invasion, tube formation and colony formation of cancer cells

**Figure 2:**
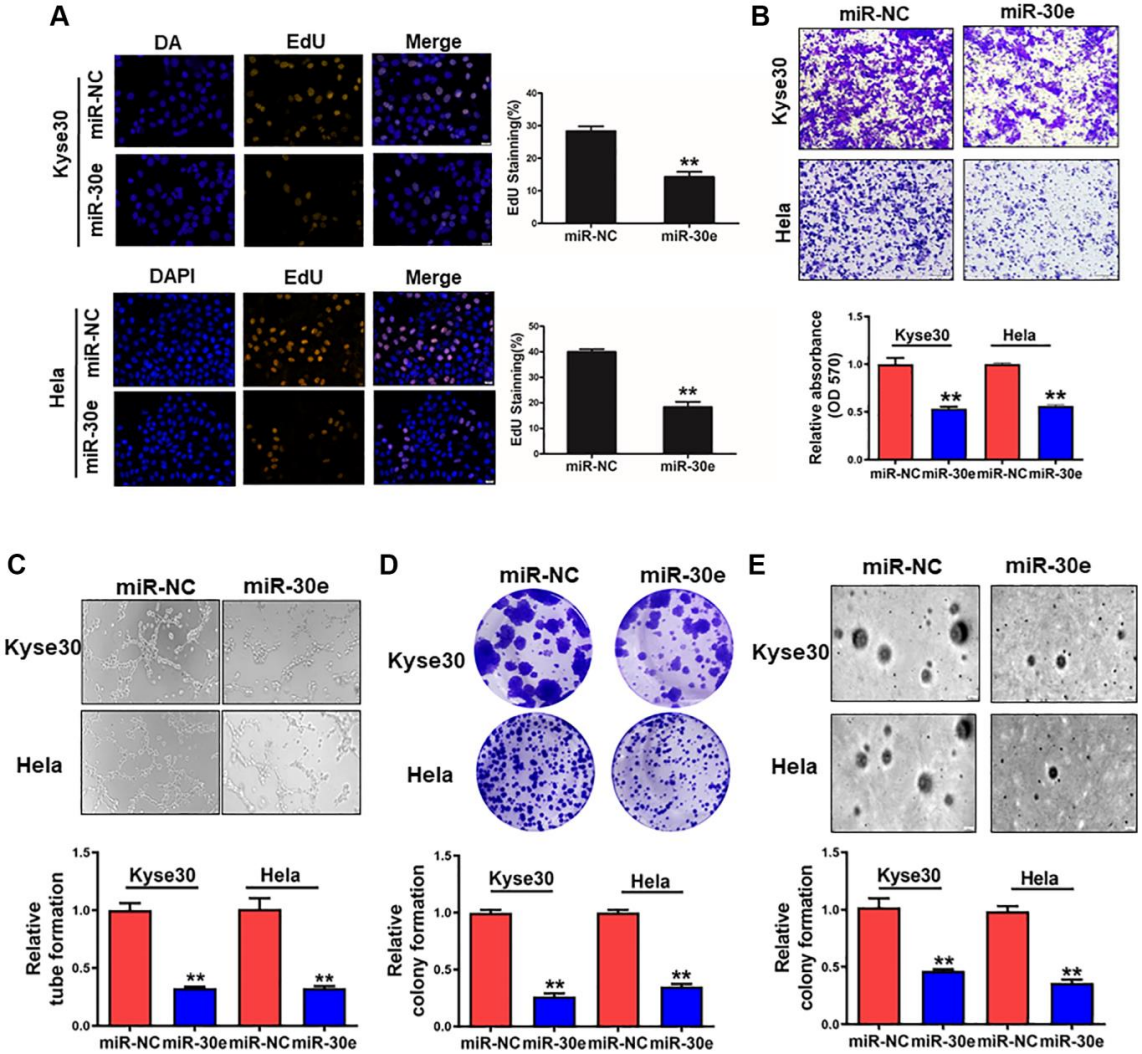
**MiR-30e overexpression inhibited cell proliferation, invasion, tube formation and colony formation of cancer cells.** (**A**) Human esophageal cancer Kyse30 cells and cervix cancer Hela cells were transfected with negative control miRNA (miR-NC) and miR-30e mimic, and the cells were cultured and stained using EdU. Immunofluorescence images of EdU, a cell proliferation marker, were analyzed in Kyse30 and Hela cells. (**B**) These cells were used to study cell invasion using the Transwell chambers with pre-coated Matrigel. (**C**) Conditioned media were collected from Kyse30 and Hela cells overexpressing miR-30e and miR-NC. HUVECs were used to test tube formation using the conditioned media obtained above. (**D**) Indicated cells were used for clonogenic assay. (**E**) Indicated cells were used for colony formation assay. Data represent mean ± SD of 3 replicates. ^**^indicates significant difference at *P* < 0.01.

To determine the role of miR-30e in cancer cells, we overexpressed miR-30e in human esophageal cancer Kyse30 cells and cervix cancer Hela cells, and found that miR-30e forced expression inhibited the proliferation of both types of cells (Figure 2A). MiR-30e overexpression also decreased the cell invasion ability of Kyse30 and Hela cells with the reduction of 50% and 40%, respectively, using Transwell assay (Figure 2B). Similarly, we found that overexpression of miR-30e inhibited tube formation, clonogenicity, and colony formation capacities (Figure 2C–2E). These results suggest that miR-30e downregulation in cancer is functionally important, which is involved in the increases of cancer cell proliferation, invasion, tube formation and colony formation.

### MiR-30e directly targeted and inhibited RPS6KB1 expression

**Figure 3:**
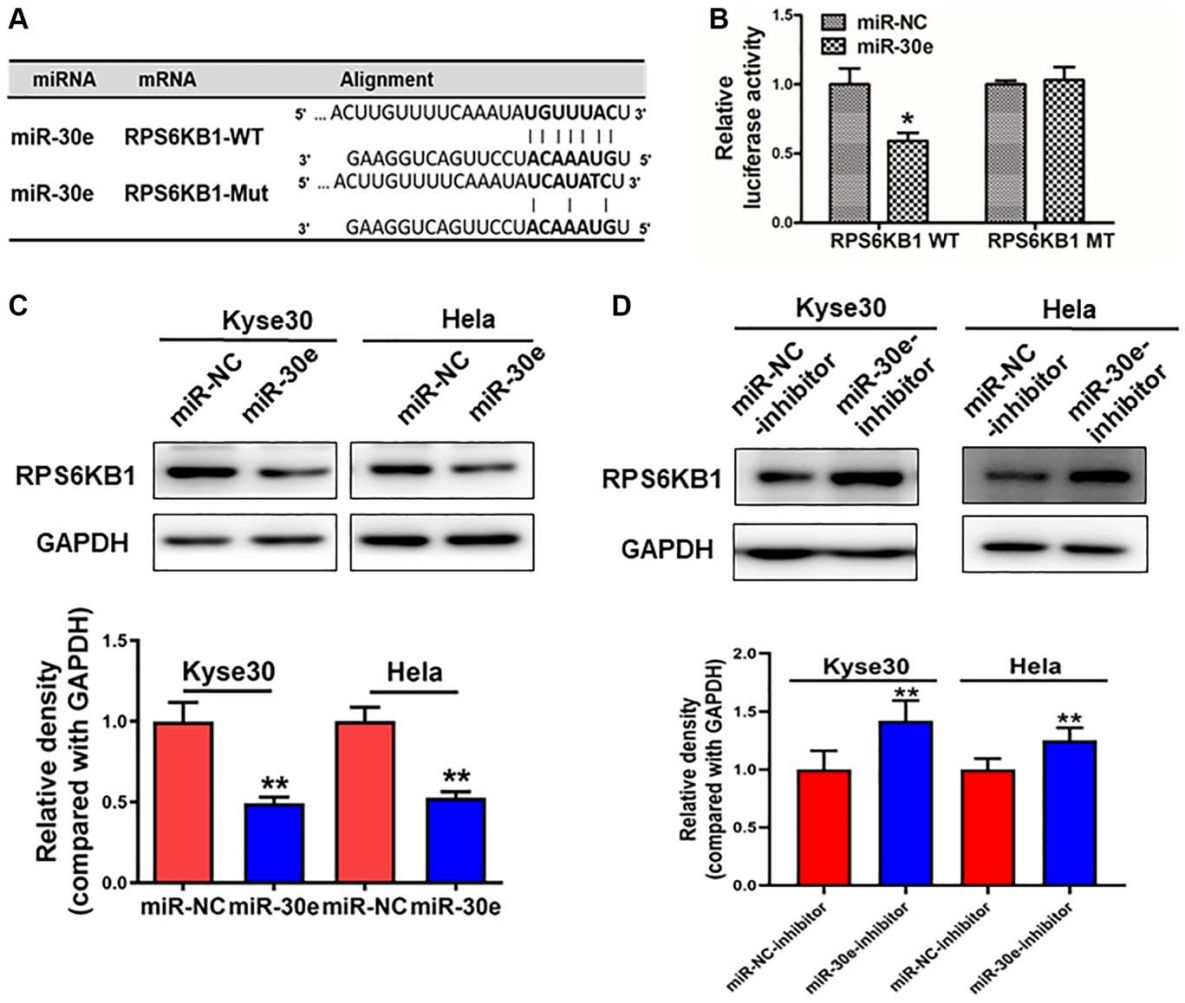
**RPS6KB1 was a novel target of miR-30e.** (**A**) The miR-30e binding site predicted in the 3′-UTR of RPS6KB1 mRNA. Mutant construct was generated at the seed sequence region of RPS6KB1 3′-UTR as indicated. The 3′-UTR fragment of RPS6KB1 mRNA containing wild-type (WT) or mutant (MT) of the miR-30e binding site was cloned into pmirGLO vector. (**B**) Kyse30 cells were transfected with pmirGLO reporter vector containing wild-type or mutant RPS6KB1 3′-UTR (indicated as RPS6KB1-WT and RPS6KB1-MT) with the combination of either miR-NC or miR-30e mimic. Luciferase activities were determined 24 h after the co-transfection. (**C**, **D**) The protein levels of RPS6KB1 in Kyse30 and Hela cells were analyzed by Western blotting assay. The density levels of RPS6KB1 were determined by ImageJ software, and relative fold changes were obtained by the ratios of RPS6KB1 to GAPDH levels. Data represent mean ±SD. of 3 replicates. ^*,**^indicates significant difference at *P* < 0.05, and at *P* < 0.01, respectively.

To elucidate the underlying molecular mechanism by which miR-30e serves as a tumor suppressor in cancer cells, we used bioinformatics tools to identify its potential target(s), and predicted that RPS6KB1 was a potential target of miR-30e (Figure 3A). In order to determine whether miR-30e directly targets RPS6KB1, 3′-untranslated region (UTR) sequences containing wide-type or mutant miR-30e binding site of RPS6KB1 were cloned into the pmirGLO vector, respectively. Kyse30 cells were co-transfected with reporter plasmids and miR-30e or miR-NC. MiR-30e transfection significantly inhibited luciferase activity of RPS6KB1 wild-type reporter, but not that of the RPS6KB1 mutant reporter with change of 4 base pairs in the binding site (Figure 3B). Western blotting assay showed that overexpression of miR-30e significantly repressed protein levels of RPS6KB1 (Figure 3C). Consistently, overexpression of miR-30e inhibitor markedly induced RPS6KB1 expression (Figure 3D). Finally, we analyzed the relationship between miR-30e and RPS6KB1 in several types of cancers (http://www.linkedomics.org/admin.php). The results (Supplementary Figure 1) showed that miR-30e expression level negatively correlated to RPS6KB1 level in invasive breast carcinoma (BRCA), glioblastoma multiforme (GBM), and lung adenocarcinoma (LUAD). Overall, the results suggest that miR-30e inhibits RPS6KB1 expression through directly targeting its 3′-UTR.

### MiR-30e overexpression inhibited tumor growth and RPS6KB1 expression, and higher RPS6KB1 expression levels were associated with lower esophageal cancer survival rate

**Figure 4:**
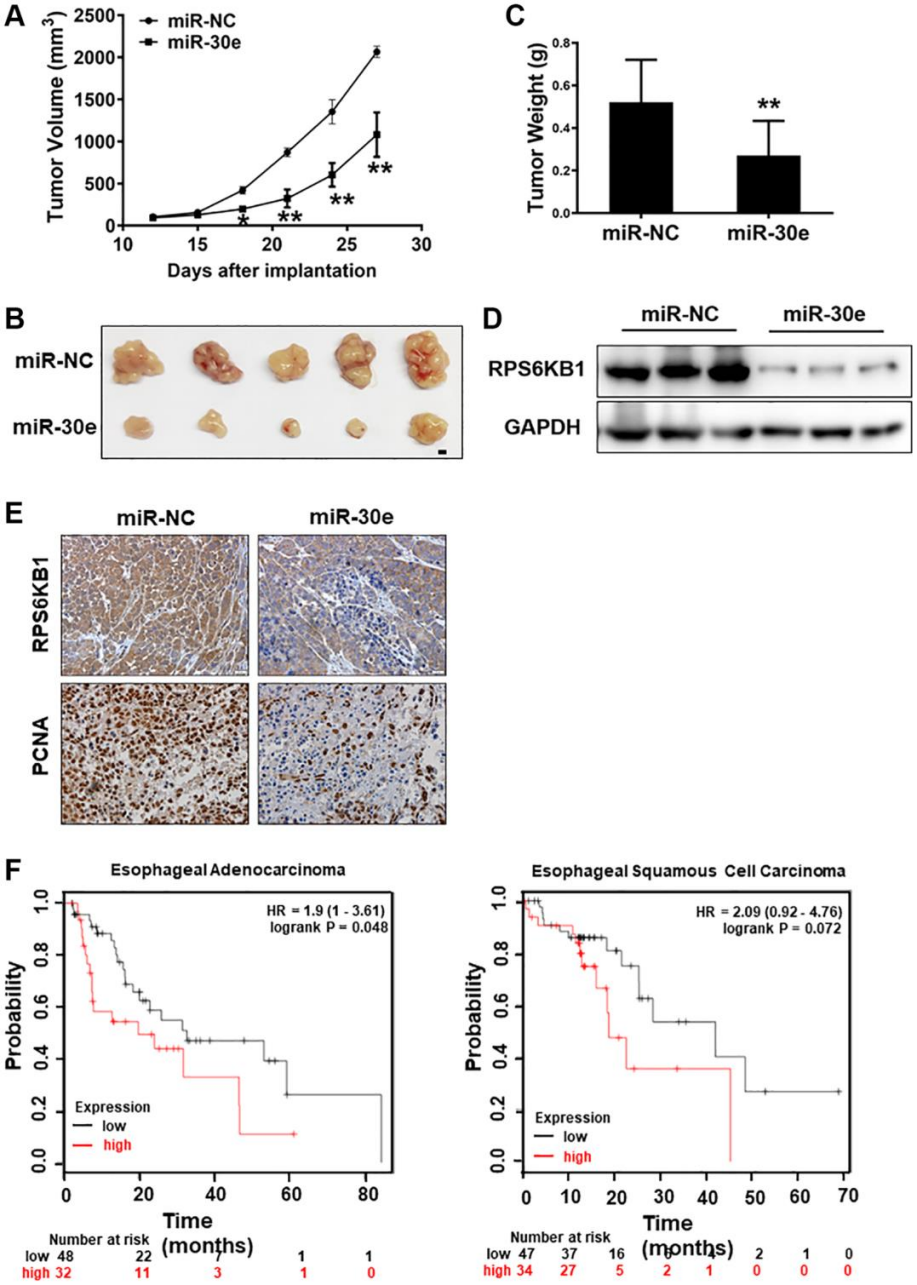
**MiR-30e inhibited tumor growth and RPS6KB1 expression, and higher levels of RPS6KB1 were associated with lower esophageal cancer survival rate.** (**A**) Kyse30 cells were infected with lentivirus expressing miR-30e or miR-NC, and cells stably expressing miR-NC and miR-30e termed as Kyse30/miR-NC and Kyse30/miR-30e were obtained after puromycin selection. Cells (5 × 10^6^ cells) in 100 μl of serum-free DMEM medium were subcutaneously injected into posterior flanks on both sides of the nude mice (*n* = 5). Tumors were measured every three days after they were visible starting on Day 12. Tumor volumes were calculated using the following formula: volume = 0.5 × (length × width^2^). (**B**) Representative pictures of tumors. Bar = 2 mm. (**C**) The weights of tumors from miR-30e and miR-NC groups were measured. (**D**) The total proteins were extracted from xenografts and subjected to Western blotting analysis to test levels of RPS6KB1. GAPDH levels were used as an internal control. (**E**) The expression levels of RPS6KB1 and PCNA in tumor tissues were analyzed by immunohistochemical (IHC) staining. (**F**) The Kaplan Meier plotter was used to detect the correlation between overall survival (OS) and RPS6KB1 expression levels in esophageal adenocarcinoma and esophageal squamous cell carcinoma tissues, respectively. Data represent mean ±SD of 3 replicates. ^*,**^indicates significant difference at *P* < 0.05 and at *P* < 0.01, respectively.

To further understand the role of miR-30e in tumor growth, Kyse30 cells were transduced by lentivirus expressing miR-30e or negative control (miR-NC). After the selection of cells in the presence of puromycin for 2 weeks, cell lines stably expressing miR-NC and miR-30e, termed as Kyse30/miR-NC and Kyse30/miR-30e respectively, were established and confirmed. The cells were injected into posterior flanks of immune-deficient mice, and tumor sizes were measured starting on Day 12 after implantation. Compared to miR-NC group, miR-30e-expressing group developed significantly smaller tumors from Day 12 to Day 27 (Figure 4A–4C). Consistent with tumor growth results, the protein levels of RPS6KB1 in tumor tissues from miR-30e overexpression group were much lower than those from miR-NC group analyzed by Western blotting assay (Figure 4D). Moreover, the xenografts from miR-30e-overexpressing Kyse30 cells showed lower expression levels of PCNA by IHC staining compared to control (Figure 4E), demonstrating that forced expression of miR-30e inhibited tumor cell proliferation. To further determine whether the expression of miR-30e novel target RPS6KB1 is relevant with human esophageal cancer, we studied expression levels of RPS6KB1 in the cancer tissues and the cancer outcome. We found that higher RPS6KB1 expression levels were strongly associated with lower overall survival rate of esophageal adenocarcinoma and esophageal squamous cell carcinoma patients by Kaplan Meier plotter analysis (Figure 4F). Taken together, these results suggest that miR-30e suppresses tumor growth and inhibits RPS6KB1 expression *in vivo*, and RPS6KB1 acts as an oncogene in esophageal cancer.

### Analysis of RPS6KB1 and miR-30e expression levels with overall survival and tumor progression in several types of cancer

**Figure 5:**
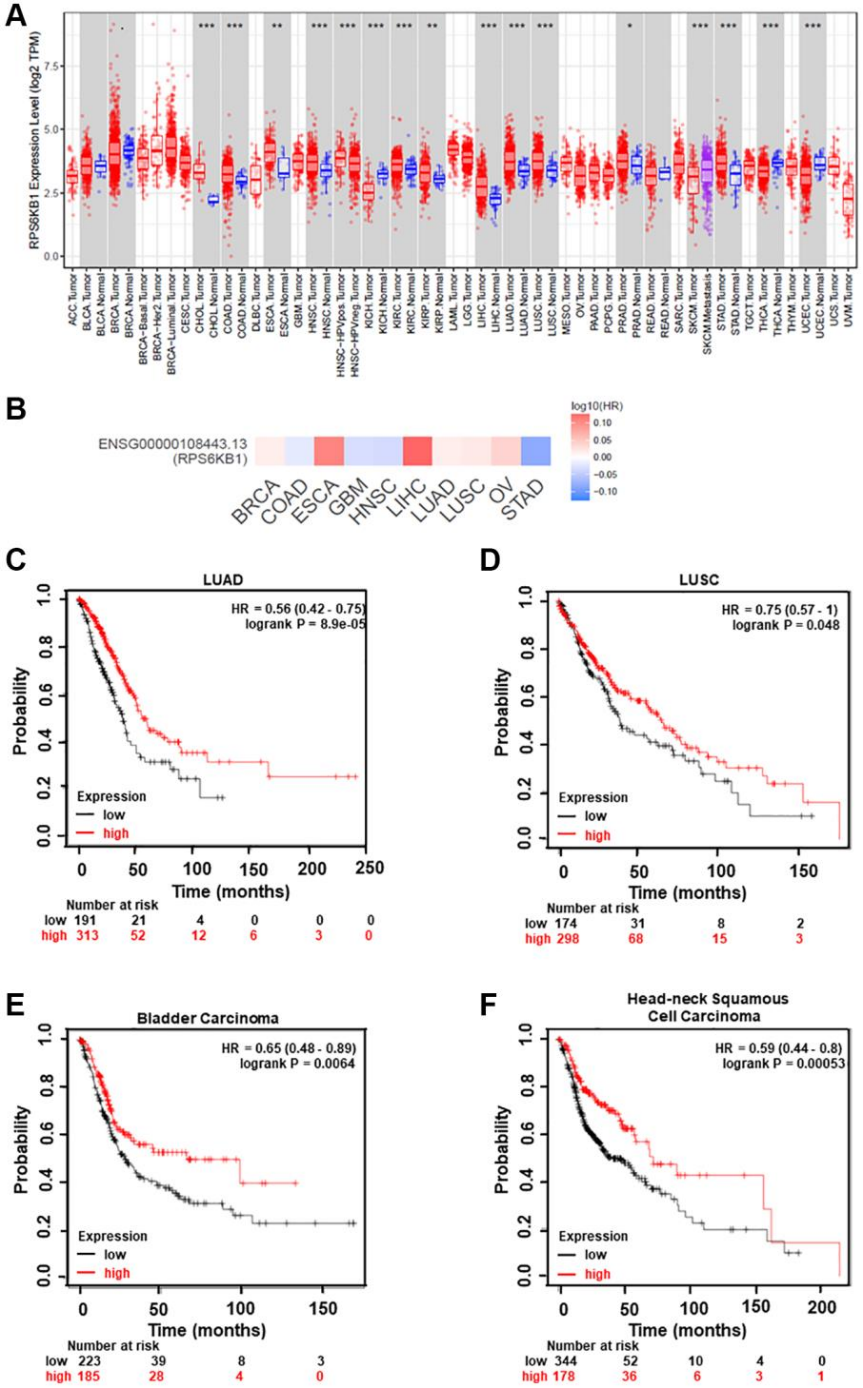
**Analysis of RPS6KB1 and miR-30e expression levels, overall patient survival rate and tumor progression in several types of cancer.** (**A**) Human RPS6KB1 levels in different types of tumor from TCGA were determined using TIMER database (http://timer.comp-genomics.org/), and RPS6KB1 expression is upregulated in most types of tumor. (**B**) GEPIA (http://gepia.cancer-pku.cn/index.html) was used to analyze the prognostic value of RPS6KB1 expression based on the log-rank test in 10 types of cancer. (**C**–**F**) Kaplan-Meier plotter (http://kmplot.com/analysis/), an online database of published microarray datasets, was used to analyze the correlation between miR-30e expression levels and overall patient survival in lung adenocarcinoma (LUAD), lung squamous cell carcinoma (LUSC), bladder carcinoma and head and neck squamous cell carcinoma.

To determine whether RPS6KB1 expression levels are upregulated in tumor tissues of other types of cancer, we examined RPS6KB1 RNA levels in various tumor tissues and adjacent healthy tissues using the RNA sequencing data obtained from the TCGA database (TIMER database, http://timer.comp-genomics.org/). The results showed that RPS6KB1 levels were upregulated in most types of cancer, including colon adenocarcinoma, esophageal carcinoma, head and neck squamous cell carcinoma, lung adenocarcinoma, lung squamous cell carcinoma, stomach adenocarcinoma (Figure 5A). We also used the GEPIA database (http://gepia.cancer-pku.cn/index.html) to investigate whether RPS6KB1 expression levels correlated with the prognosis of cancer patients, and fount that RPS6KB1 expression levels significantly impacted the prognosis of seven types of cancer, including breast, lung, ovarian and liver cancers (Figure 5B). In order to determine whether miR-30e expression levels are correlated with cancer overall survival rate, we found that levels of miR-30e expression were inversely correlated with the patient survival in lung adenocarcinoma (LUAD), lung squamous cell carcinoma (LUSC), bladder carcinoma and head and neck squamous cell carcinoma using the Kaplan-Meier plotter (http://kmplot.com/analysis/), showing that higher miR-30e levels were associated with better patient outcome (Figure 5C–5F).

## DISCUSSION

It is well-known that microRNAs alterations affect expression of many genes that are important in the initiation, maintenance and progression of human cancers [10, 11, 27]. In this study, we found that miR-30e expression was greatly downregulated in EC patients. In the search of molecular mechanism of miR-30e suppression, we found that RPS6KB1 is a direct novel target of miR-30e, which may be important for tumor development. RPS6KB1 has been identified as an integrator of nutrient and growth factor signals for key cellular processes such as cell proliferation, invasion, and angiogenesis [22, 23]. In protein synthesis, it plays an important role by interacting with or activating other transcription factors [28]. Our results demonstrated that forced expression of miR-30e dramatically suppressed RPS6KB1expression at the protein level in human esophageal cancer cell line Kyse30 and human cervical cancer cell line HeLa. Growing evidence shows that RPS6KB1 overexpression/activation is a critical driver of initiation and progression of tumors, and that the inactivation of RPS6KB1 may be therapeutically effective in many types of cancer [29–32].

Recent studies have reported that the gain of function is the central role of RPS6KB1 in resistance to tyrosine kinase (TKI) in lung cancer, and its specific inhibitor PF-4708671 shows synergistic effect to enhance TKIs efficacy to suppress tumor growth *in vivo* [33]. Several studies have shown that single reagent treatment often fails in clinical practice, additional studies are necessary to optimize combination therapies that include RPS6KB1 inhibitor to treat EC. Our results here showed that miR-30e suppression and RPS6KB1 upregulation were important in cell migration, colony formation and tumor growth of cancer cells. We would like to see if miR-30e/RPS6KB1 pathway would be new target for EC clinical therapy by using combination of cisplatin/pacilitaxel and miR-30e or RPS6KB1 inhibitor PF-4708671 in the future.

In conclusion, miR-30e was identified as a novel tumor suppressor of EC. We further demonstrated that miR-30e directly targeted RPS6KB1 by binding to its 3′-UTR and downregulation of miR-30e was implicated in the progression of EC via RPS6KB1 overexpression. More importantly, TCGA clinical evidence revealed that both miR-30e and RPS6KB1 levels could be used to predict prognosis and survival rate of EC patients. These findings suggest the functional importance of miR-30e/RPS6KB1 axis, and potential clinical application by altering this pathway as new therapeutic option for human EC therapy in the future.

## MATERIALS AND METHODS

### Cell culture and reagents

Human esophagus cancer Kyse30 cells, human cervical cancer HeLa cells and human embryonic kidney 293T cells were purchased from ATCC (Manassas, Virginia, USA). Kyse30 cells were cultured in RPMI 1640 medium (Gibco), and HeLa and 293T cells were cultured in Dulbecco’s modified Eagle’s medium (DMEM; Gibco), with supplement of 10% fetal bovine serum (FBS) and 100 U/mL penicillin, 100 μg/ml of streptomycin (ThermoFisher Scientific, Waltham, MA). Cells were maintained in a 37°C incubator with 5% CO_2_.

### Esophageal cancer specimens

Human esophageal cancer samples and adjacent normal samples were obtained in the tissue bank of the Linzhou Cancer Hospital, Henan, China. All patients received informed written approval. The study was approved by the Zhengzhou University Ethics Committee. All tissue samples in the tissue bank of the institute were from the patients who had undergone surgery and frozen immediately in liquid nitrogen before analysis. The samples we used for these experiments were obtained without patient information from the tissue bank.

### EdU proliferation assay

The relative proliferation rates of cancer cells Kyse30 and HeLa were detected and analyzed with EdU Imaging Kits using 5-ethynyl-2′-deoxyuridine (EdU, from Riobio, China). Cells were seeded onto Millicell^®^ EZ SLIDE 8-well Slides (MilliporeSigma, Burlington, MA) in medium with 10% FBS for 2–3 days. After incubated with 10 μM EdU, cells in each well were fixed. Permeability and DNA spotting were performed in accordance with the protocol of the manufacturer. Fluorescence microscopy was used to determine the proportions of cells that incorporated EdU.

### Clonogenic and colony formation assays

For clonogenic assay to test the ability of a single cell to clone itself and grow into a full colony, single cells at 300 cells/well were seeded in 6-well plate and cultured for about 2 weeks. Cells were washed, fixed and stained with 0.05% violet crystal (Millipore Sigma, Burlington, MA), and washed by 10% methanol at room temperature, and the pictures were taken by a camera. For colony formation assay, single cells (300–500 per well) were seeded in 6-well plate using SeaPlaque agar with medium and cultured for 2–3 weeks. Then cells were washed, stained with 0.01% violet crystal, and washed by water at room temperature. The pictures were taken using Nikon inverted microscope (Nikon Instruments Inc., Melville, NY).

### Cell invasion assay

1 × 10^5^ Kyse30 and HeLa cells in serum-free medium were seeded into Matrigel pre-coated Transwell upper chamber (BD Bioscience, San Joes, CA) for cell invasion analyses, and the complete medium with 10% FBS was used as chemoattractant in the lower chamber. After 18–24 hours of incubation, cells were stained with 0.05% crystal violet in 10% methanol. Cells that didn’t go through the pores were carefully removed with cotton swab, then spotted cells were imaged and counted using a microscope (Nikon Instruments Inc., Melville, NY).

### Tube formation assay

Tube formation assay was performed as we previously described [34]. Briefly, conditioned media from miR-NC or miR-30e treated cells were collected. 2 × 10^4^ HUVEC cells were suspended in the conditioned media and seeded into Matrigel pre-coated 96-well plate. The formation of tube-like structures was detected after 6–12 hours. The length of tubal structures was counted using Image J software. The results were expressed as the medium ± SD from three independent experiments.

### RNA extraction, RT-PCR, and real-time quantitative PCR

The total RNAs were extracted and purified from cancer cells and human tissues using TRIzol reagent, as specified by the manufacturer (Thermo Fisher Scientific, Waltham, MA). RT SuperMix reactions to qPCR were carried out according to the manual (Vazyme, China). The miR-30e qRT-PCR Primer Kit (Riobio, China) was used to measure expressive levels of miR-30e, and U6 expression was used as an internal control. The quantitative RT-PCR on a QuantStudio 5 (Applied Biosystems) system was used to calculate the fold changes by using SYBR Green Mix (Vazyme, China), with relatively quantified methodology (2^–ΔΔCt^).

### Protein extraction and immunoblotting

In an ice-cold radioimmunoprecipitation assay (RIPA) complemented with protease inhibitors, cells or human tissue were lysed for 30 minutes, then centrifuged at 13,000 rpm at 4°C for 15 minutes. The protein concentration was determined using a bicinchoninic acid test and SDS-polyacrylamide gel electrophoresis was used to analyze the protein extracts. The transfer was performed in the transfer buffer (20 mM Tris, 150 mM Glycin, 20% methanol). The nitrocellulose membranes were blocked with 5% non-fat milk for 2 hours prior to overnight incubation by first antibodies against RPS6KB1 (Cell Signaling Technology, Danvers, MA) and GAPDH (Bioworld, Nanjing, China). The membranes were incubated by secondary antibodies for 2 hours, washed, and protein bands were visualized by the electrochemiluminescence detection system (Thermo Fisher Scientific).

### Luciferase reporter assay

The pmirGLO vector (Promega, Madison and WI) was used to insert specific fragments of miRNA target gene, between SacI and XhoI restriction sites, which included a wild-type or a mutant binding site of miR-30e. The cells were cultured in 24-well plates and transfected using Lipofectamine 3000 (Thermo Fisher Scientific, Waltham, MA) according to instructions by the manufacturers, with either wild type (WT) or mutant type (mut) luciferase reporter plasmid and equal amount of miR-30e mimic or miRNA negative control (miR-NC). The luciferase activities were analyzed using the Dual Luciferase Reporter Assay System (Promega, Madison, WI) 24 hours after transfection.

### Tumor grow assay

Six-week-old BALB/c (nu/nu) mice were purchased from the Vital River Laboratory Animal Technology (Beijing, China) and maintained under specific pathogen free conditions at Zhengzhou University. Mice were randomly divided into two groups (5 mice/group) and subcutaneously injected 5 × 10^6^ Kyse30/miR-NC or Kyse30/miR-30e cells to form tumors. Mice were monitored 3 times/week for tumor development and the size of tumors was measured with calipers. Tumor volumes were calculated according to the formula (width^2^ × length)/2. The mice were sacrificed, xenografts were trimmed out and photographed on Day 28 post-implantation. Tumor sections were subjected to immunohistochemical staining utilizing RPS6KB1 and PCNA antibodies. The animal studies were approved by the Animal Care and Use Committee at Zhengzhou University, and all animal care and methodologies were in accordance with institution guidelines.

### Statistical analysis

Data was shown as means ± SD from three replicates and the results were analyzed using GraphPad Prism 8 (La Jolla). The student’s *t*-test was used to analyze the quantitative variables between two groups with significant differences at *P* < 0.05.

## Supplementary Materials

Supplementary Figure
