# Supplementary material for: MiRNA-30e downregulation increases cancer cell proliferation, invasion and tumor growth through targeting RPS6KB1
Source: Aging (Albany NY). 2021 Nov 2;13(21):24037–49. doi: 10.18632/aging.203665 (PMC8610128; doi:10.18632/aging.203665)
Supplement: Supplementary Figure [file aging-13-203665-s001.pdf]

SUPPLEMENTARY FIGURE

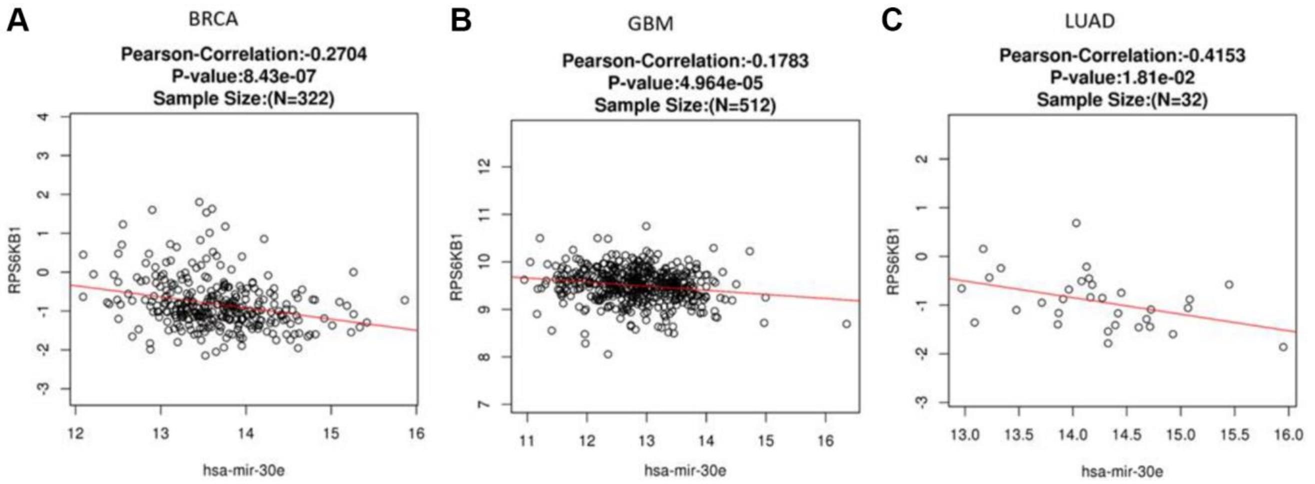

Supplementary Figure 1. Negative correlation between miR-30e and RPS6KB1 in various types of cancers. We analyzed the relationship between miR-30e and RPS6KB1 in several types of cancers (http://www.linkedomics.org/admin.php). The results showed that there was an inverse relationship between miR-30e and RPS6KB1 in breast invasive carcinoma (BRCA) (**A**), Glioblastoma multiforme (GBM) (**B**), and lung adenocarcinoma (LUAD) (**C**).
